# Editorial: Knee arthroplasty: techniques and complications

**DOI:** 10.3389/fsurg.2023.1202714

**Published:** 2023-05-16

**Authors:** Sujit Kumar Tripathy, Senthil Sambandam, Jibanananda Satpathy, Stanislav Bondarenko

**Affiliations:** ^1^ Department of Orthopaedics, All India Institute of Medical Sciences, Bhubaneswar, Bhubaneswar, India; ^2^ Dallas VA Medical Center, United States Department of Veterans Affairs, Dallas, TX, United States; ^3^ Department of Orthopaedics, Virginia Commonwealth University Health System, Richmond, VA, United States; ^4^ Sytenko Institute of Spine and Joint Pathology, Academy of Medical Science, Kharkiv, Ukraine

**Keywords:** total knee anthroplasty, prosthetic joint infection (PJI), complication, nomogram, PEEK=polyetheretherketone, arthroplasty

**Editorial on the Research Topic** Knee arthroplasty: techniques and complications

Knee arthroplasty is considered the most successful surgery of the past decade. Even then, 10%–20% of patients remain dissatisfied following the procedure (1). The understanding of this technique has developed from mechanical to kinematic alignment, and the accuracy has been enhanced from conventional manual technique to computer navigation, patient-specific implantation (PSI), and robotic-assisted surgery. Using various parameters, researchers are trying to predict the complications, such as fever, infection, loosening, and hospital stay. The collection of articles in this special issue has published many such innovative concepts that will be helpful to arthroplasty surgeons.

The restricted kinematic alignment (RKA) restores the pre-arthritic constitutional lower limb alignment in TKA Vendittoli et al. The principles aim at hip-knee-ankle (HKA) angle restoration of 3 degrees on either side, joint line obliquity within 5 degrees of neutral, restoration of knee soft tissue tension, preservation of femoral anatomy, and making the pivot point on the less damaged condyle. As nearly half of the patients need anatomic modifications to fit within the restricted kinematic alignment boundaries, the technique can be best adopted using PSI, computer navigation, or a robot. The clinical report suggested better mid-term function, a higher patient-reported outcome score, and a gait pattern equivalent to a non-arthritic limb. Unlike mechanical alignment, RKA avoids extensive corrections and soft tissue releases. RKA can also be applied to revision TKA, particularly in early, non-wear-related, unsuccessful mechanically aligned TKAs Kostretzis et al.

The article “Clinical and Radiological Changes of the Ankle in Knee Osteoarthritis with Varus After Total Knee Arthroplasty: A Systematic Review” provides a comprehensive overview of the clinical and radiological changes that occur in the ankle joint and hind foot following total knee arthroplasty (TKA) in patients with knee osteoarthritis and varus alignment Feng et al. The authors systematically reviewed the literature and analyzed the results of 8 studies involving 913 patients with 1,157 knees. TKA in patients with varus knee osteoarthritis significantly improved ankle function and hindfoot alignment. However, the ankle pain increased after TKA in patients with osteoarthritis ankle, hindfoot stiffness, and residual knee deformity. The authors suggested that patients with residual knee deformity and pain in the hindfoot should receive treatment for the foot problem after six weeks of TKA as further improvement is remote. The authors concluded with a message to the arthroplasty surgeons that all patients should have thorough clinical and radiological examinations of the ankle/hindfoot before TKA. Symptomatic ankle or hindfoot problems with radiological changes can receive treatment in the preoperative or postoperative periods of TKA. However, patients without ankle/hindfoot problems before TKA may also develop foot symptoms after TKA, and they can be treated when discomfort occurs.

Noninfectious fever (NIF) after TKA is a serious issue for orthopedic surgeons. It is defined as a raised body temperature equal to or higher than 38.0°C with a negative bacterial culture of blood, urine, sputum, and synovial fluid aspirate. It leads to prolonged antibiotic use and hospital stay, thus increasing the medical cost of treatment. The retrospective data published in this special issue reported a 39% incidence of NIF in 146 patients of TKA Xu et al. The proposed nomogram by the team predicted NIF within seven days of TKA from three parameters:

• Intraoperative blood loss
• The volume of postoperative drainage fluid
• Frequency of blood transfusion

The author proposed that this nomogram had a sensitivity of 54% and a specificity of 82%. While the nomogram can predict fever of noninfectious origin, the study suggests surgeons pay more attention to perioperative bleeding control measures and judicious use of blood transfusion to minimize the incidence of NIF.

An exciting article predicted the length of hospital stay after TKA from a nomogram Liu et al. The parameters were age, Hb, surgical duration, procedure description, diabetes mellitus, day of operation, blood transfusion, and repeat surgery within 30 days. Three quartiles of patients (Q3) had a hospital stay of six days, and 20% had an extended stay.

In an innovative model, the Polyetheretherketone (PEEK) based TKA allowed computed tomographic assessment of the underlying bone with bone mineral density (BMD) measurement Cai et al. The authors noted a reduction in BMD in a lateral tibial plateau at three months follow up and noted tibial prosthesis overhang in 2 of 10 patients. PEEK may offer the advantages of accurately measuring periprosthetic bone changes and prosthesis overhang while avoiding metal-related reactions.

Prosthetic joint infection (PJI) is alarming as the incidence is increasing because of increased primary TKA. The available serum biomarkers lack specificity, and synovial fluid markers play a more significant role in diagnosis (2). The prospective study on PJI reported that both synovial fluid IL-4 (SF-IL4) and polymorphonuclear cell percentage (SF-PMN %) could diagnose PJI with 97% specificity and 96% accuracy when the cut-off values were 1.7 pg/ml and 75%, respectively Huang et al.

The data from Richmond, USA, about stem survivorship in revision TKA (*n* = 133 revision in 122 patients) revealed that hybrid (primary fixation through diaphyseal stem-bone fixation and secondarily with epiphyseal or metaphyseal cement) and fully cemented techniques of both femoral and tibial stem implantations have similar survival rates Kemker et al. The stem survivorship was 81% in the hybrid group and 85% in the cemented groups at a minimum follow-up of 2 years. The authors did not find an association between age, sex, and BMI with the failure in either group. There were no differences in length of hospital stay and hemoglobin drop in both the groups suggesting no difference in early postoperative complications rate.

The technique of fusiform capsulectomy of the posterior capsule and percutaneous flexion tendon release for TKA in the bony ankylosed knee with severe flexion contracture (>80°) has shown promising results without neurovascular complications or infection. At medium-term follow-up, the authors reported a significantly improved knee society score with a mean range of motion of 100 degrees Chen et al.

The articles in this special issue are blended with research articles, new technology, and systematic review. Information provided through these articles will be helpful to arthroplasty surgeons for better patient care, precision in surgery and future development.
